# Pure Organic Active Compounds Against Abiotic Stress: A Biostimulant Overview

**DOI:** 10.3389/fpls.2020.575829

**Published:** 2020-12-23

**Authors:** Ana L. García-García, Francisco J. García-Machado, Andrés A. Borges, Sarai Morales-Sierra, Alicia Boto, David Jiménez-Arias

**Affiliations:** ^1^Grupo de Agrobiotecnología, Departamento de Ciencias de la Vida y de la Tierra, Instituto de Productos Naturales y Agrobiología, Consejo Superior de Investigaciones Científicas, San Cristobal de La Laguna, Spain; ^2^Grupo Síntesis de Fármacos y Compuestos Bioactivos, Departamento de Química de Productos Naturales y Sintéticos Bioactivos, Instituto de Productos Naturales y Agrobiología, Consejo Superior de Investigaciones Científicas, San Cristobal de La Laguna, Spain; ^3^Universidad de La Laguna, San Cristóbal de La Laguna, Spain

**Keywords:** Biostimulant, abiotic stress, amino acid, polyamine, biopolymer, vitamin, melatonin

## Abstract

Biostimulants (BSs) are probably one of the most promising alternatives nowadays to cope with yield losses caused by plant stress, which are intensified by climate change. Biostimulants comprise many different compounds with positive effects on plants, excluding pesticides and chemical fertilisers. Usually mixtures such as lixiviates from proteins or algal extracts have been used, but currently companies are interested in more specific compounds that are capable of increasing tolerance against abiotic stress. Individual application of a pure active compound offers researchers the opportunity to better standarise formulations, learn more about the plant defence process itself and assist the agrochemical industry in the development of new products. This review attempts to summarise the state of the art regarding various families of organic compounds and their mode/mechanism of action as BSs, and how they can help maximise agricultural yields under stress conditions aggravated by climate change.

## Introduction

The United Nations has set 17 goals for sustainable development worldwide, number two being to reach zero hunger by 2030. To achieve it, one of the suggested strategies is to double agricultural production. Food demand is expected to increase by 100–110% by 2050 (Tilman et al., 2011), but some studies indicate that yield trends are insufficient to reach this goal (Ray et al., 2013). One of the reasons is climate change. According to the IPPC report, a major drop in crop yields is expected worldwide with a global warming of 2°C, with a high confidence level (Hoegh-Guldberg et al., 2018). This report also points to a reduction in the nutritional quality of cereal crops as the temperature rises. In fact, one of the most important challenges is feeding a growing population that will reach 9 billion by 2050 (Tilman et al., 2011) in a climate change scenario. Furthermore, during the 21st century, heat waves, heavy precipitations and sea-level rises are forecast, with subsequent droughts, floods and salinity among the most critical direct consequences affecting food production (Mba et al., 2012). These are already having serious effects on human health and social well-being. Nowadays, abiotic stresses in plants are the main cause of severe yield losses of 50–80%, depending on the crop and geographical location (Zhang et al., 2018).

**FIGURE 1:**
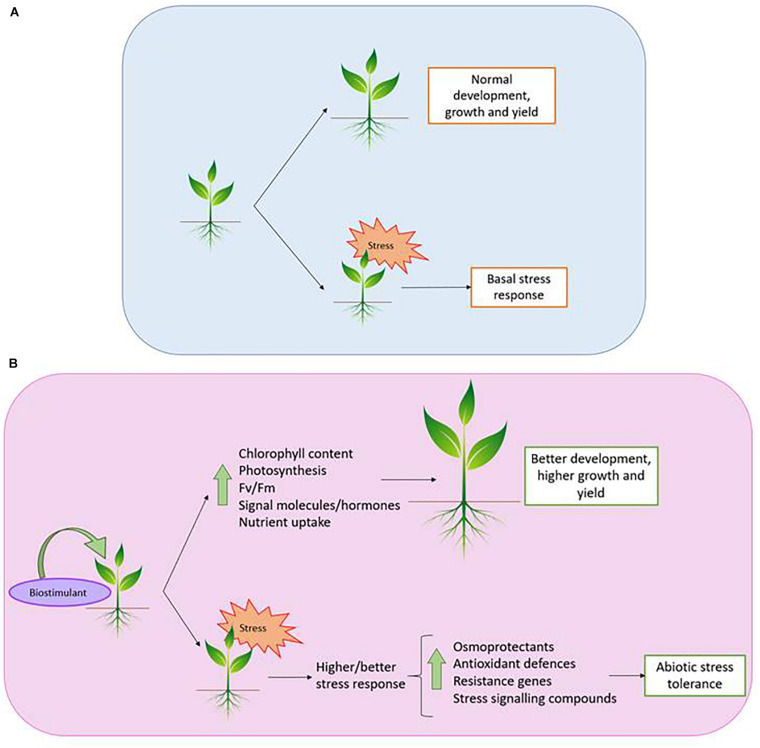
Plant response against stress. Without **(A)** and with biostimulant **(B)**.

This daunting situation provides an excellent opportunity for plant scientists to apply their knowledge in the agricultural field, in the attempt to increase productivity under abiotic stress. In this regard, a promising strategy is the use of biostimulants (BSs; Bulgari et al., 2019), since they promote plant growth and improve crop productivity without negative impacts on the environment (Figure 1), therefore allowing the reduction in the use of chemical fertilisers (Singh et al., 2018; Xu and Geelen, 2018). It should be pointed out that BSs act solely as triggers for plant natural defenses, and furthermore, only small amounts are needed to increase tolerance against various stresses (Figure 1), which is quite interesting for commercial purposes.

The concept of BSs has evolved over time (European Biostimulants Industry Council [EBIC], 2020; du Jardin, 2012, 2015; Traon et al., 2014; Halpern et al., 2015; Yakhin et al., 2017). The current definition of plant BSs by the EU regulation (2019) is: “*A product that stimulates plant nutrition processes independently of the product’s nutrient content, with the sole aim of improving one or more of the following characteristics of the plant or the plant rhizosphere: (a) nutrient use efficiency; (b) tolerance to abiotic stress; (c) quality traits; or (d) availability of confined nutrients in the soil or rhizosphere.*”

On the other hand, du Jardin (2015) defined it as any compound or microorganism used to enhance plant growth, stress response and/or crop quality, regardless of its nutrients content. Later, the same author pointed out that academic, regulatory and corporative entities agree that BSs are modulators of life processes in plants that enhance growth and resource use efficiency, under stress or non-stress conditions (du Jardin et al., 2020).

BSs comprise a wide range of compounds, from amino acids or amines to biopolymers. Therefore, there are different proposals to classify these compounds, as discussed in the review by Yakhin et al. (2017). However, the current classification was introduced by du Jardin (2015) and is based on the source of the biostimulant, even if this approach does not always provide adequate information on its biological activity (Bulgari et al., 2019). Thus, du Jardin established seven categories: humic and fulvic acids, seaweed and botanical extracts, protein hydrolysates and *N*-containing compounds, chitosan and other biopolymers, inorganic compounds and beneficial fungi and bacteria.

Most of the work in this field has been carried out with complex product mixtures, such as plant or seaweed extracts, recycled waste products, protein hydrolysates and so on. In part, this is due to the fact that a combination of several useful compounds (polymers, amino acids, vitamins, minerals) with different modes of action can be more effective than the use of a pure active principle, especially if the compounds act in a synergistic way (Bulgari et al., 2019). Another reason is the “circular-economy,” since the processing of low-cost natural resources or waste usually produces mixtures (du Jardin et al., 2020), from which the identification and isolation of active principles is costly, in time and material resources, particularly because these active principles are usually present in small amounts.

However, the use of mixtures presents some problems, as commented by Yakhin et al. (2017). The first is the homogeneity of different batches, which can affect the interaction of the product with the environment and, therefore, the results in the crops. For instance, plants and seaweeds used as source of biostimulants may vary in their contents according to their development stage, period of the year, environmental conditions, and even competition or interactions with other organisms. To overcome this problem, many companies attempt to collect and process natural resources (or waste by-products) under carefully controlled conditions, and also to analyse the final products. However, it is not always possible to guarantee a perfectly standardised production protocol, and if the active principles are scarce or partially unknown, the analysis is problematic (Yakhin et al., 2017). Even when the active ingredient is difficult to isolate, efforts are being made to find the purest active fractions. Thus, in the area of humic substances, it isnoteworthy that recently quantitative QSAR for humic substances has been reported (Savy et al., 2020), but at the moment effects in plants have not been explored, remaining out of the aim of this review.

Moreover, products with a pure active compound present advantages over extracts and other mixtures, since it is easier to determine not only their physiological effect and mode of action but also their mechanism of action, thus simplifying the certification and registration processes (Yakhin et al., 2017). For this reason, different companies are trying to develop new BSs based on the most effective active molecules (Bulgari et al., 2019). Among the pure compounds found as the basis of formulations to promote plant growth under stress are melatonin (Arnao and Hernández-Ruiz, 2019), GABA (Li et al., 2017a), or menadione sodium bisulphite (MSB; Borges et al., 2014), among others.

**FIGURE 2:**
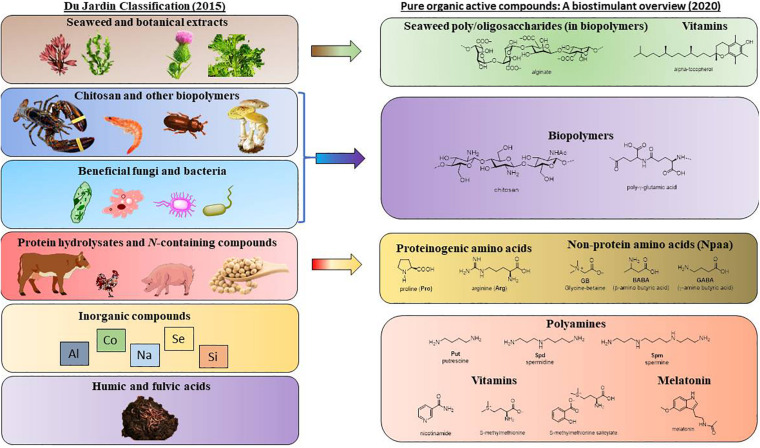
Pure organic active compounds and their relationship with biostimulants classified by du Jardin (2015).

Comparing with the DuJardin classification (Figure 2), humic acids are not treated in this review either because the exact chemical structures were not provided, or because the compounds in the purified fractions were not described for protection against abiotic stresses. Protein hydrolysates and extracts from seaweed, terrestrial plants or microorganisms, as well as recycled waste, are complex mixtures outside the scope of this review, but many individual components isolated from these mixtures are described herein. Commercial formulations based on many ingredients, or with poorly described composition (industrial secret) are not covered either. Although whole microorganisms (beneficial fungi and bacteria) are not included for the same reason, pure compounds of microbial origin, such as vitamins, amino acids, melatonin or biopolymers, are discussed in detail. For the interested reader, there are reviews on microbial biostimulats to face abiotic stress (Woo and Pepe, 2018; Van Oosten et al., 2017; Calvo et al., 2014) and the challenges posed by climate change (Sangiorgio et al., 2020; Naamala and Smith, 2020). Since the performance of microbial biostimulants can be affected by environmental variables, in the latter review and also in (Yakhin et al., 2017), the use of microbial-derived compounds is presented as an alternative.

**FIGURE 3:**
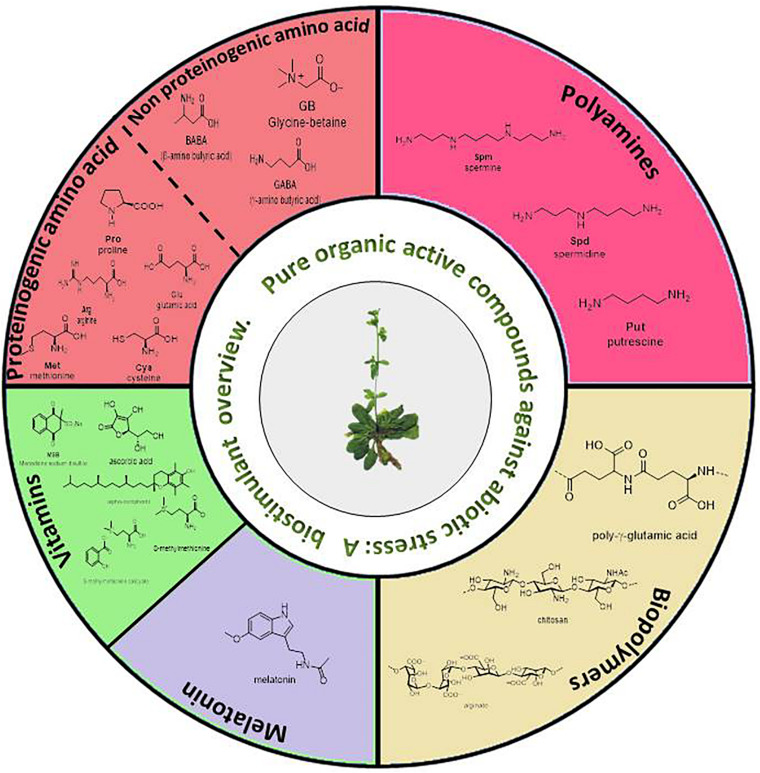
Summary of pure organic active compounds addressed in this review.

Thus, the sections developed in this review are (Figure 3): amino acids and other *N*-compounds (proteinogenic and no-proteinogenic amino acids, and polyamines), biopolymers, vitamins (including the natural-product derivatives S-methylmethionine salicylate and MSB), and melatonin.

Finally, it should be noted that biostimulant research is clearly a hot field, as evidenced by the large growth in publications reported by du Jardin et al. (2020). Moreover, global market for BSs is expected to reach 4.14 billion USD by 2025 (Madende and Hayes, 2020). We hope that this review will highlight the changes towards new formulations based on pure products (or purified fractions) and the use of increasingly standardised formulations in crop management, particularly to cope with abiotic stresses.

## Amino Acids and Other *N*-Containing Compounds

Amino acids are among the compounds most used as biostimulants. Commercial formulations based on these compounds are obtained from both plant and animal sources by chemical and enzymatic hydrolysis, their separate activity being unknown in many cases (Calvo et al., 2014; du Jardin, 2015; Popko et al., 2018). Their exogenous application allows the plant to save energy in their synthesis, thus increasing its capacity to use its resources for growth or to cope with stresses (Popko et al., 2018). The positive effects of amino acid-based BSs are well known, and these products are widely marketed (Botta, 2013; Cerdán et al., 2013; Popko et al., 2018; Kocira, 2019; Tadros et al., 2019). Most of the amino acids used are the 20 proteinogenic ones, but non-proteinogenic amino acids (npaa) can also be found. There are thousands of the latter, of which 250 are found in plants (Vranova et al., 2011). Here we take a look at some interesting research on plant treatment using a pure compound as an elicitor.

### Proteinogenic Amino Acids

Essential amino acids (leucine, isoleucine, methionine, phenylalanine, arginine, histidine, tryptophan, valine, threonine, and lysine) are synthesised only by plants, while non-essential amino acids (alanine, β-alanine, asparagine, cysteine, glutamine, aspartic acid, glycine, proline, serine, and tyrosine) are synthesised by both plants and humans (Kumar et al., 2017). However, little information is available on the effect of pure proteinogenic amino acids (Teixeira et al., 2017).

Methionine (Met) is part of stress-related proteins, among others. In fact, the foliar application of 4 mM methionine improved the yield of cowpea (*Vigna unguiculata*) under stress due to water-deficit, as well as the physiological and morphological features of the plant (Merwad et al., 2018). In a recent study (Alfosea-Simón et al., 2020). Met alone is capable of increasing tolerance to salt stress in tomatoes (*Solanum lycopersicum*) grown in hydroponic conditions. Met showed better results than other aminoacids alone or in mixtures. Another study (Akram et al., 2020) demonstrates that the application of Met is able to regulate the plant redox state and improve growth under stress, by increasing the compatible osmolite contents. Exposed Met residues in proteins can defend the macromolecule against oxidants, preventing damage to other protein residues (Luo and Levine, 2009). Met can be easily oxidised by different types of oxidizing agents (Weissbach et al., 2005; Boschi-Muller et al., 2008). The oxidation products can be reconverted into Met by methionine sulfoxide reductase (MSR; Vieira Dos Santos et al., 2005), an enzyme that controls Met redox state and is involved in defense mechanisms against stress (Rey and Tarrago, 2018).

Glutamate (Glu) was found to be effective against cold stress in rice (*Oryza sativa*) (Jia et al., 2017). However, the authors pointed out that combinations of Glu with CaCl_2_ or γ- aminobutyric acid (GABA) were more effective than glutamate alone. In addition, the foliar treatment of onion (*Allium cepa* L. cv. “Giza 20”) with Glu increased the yield and quality of the crop, but the benefits improved in combination with putrescine treatment (Amin et al., 2011). The protective effects of Glu are due to increased antioxidant protection, as shown in soybeans (*Glycine max*), where the activity of superoxide dismutase (SOD) and catalase (CAT) increased (Teixeira et al., 2017). Moreover, La et al. (2020) demonstrated that Glu improved tolerance to drought in Canola by increasing the concentration of compatible osmolites, and the levels of proline biosynthesis genes.

Proline (Pro) is probably the most widely used amino acid to prevent losses due to abiotic stress. There are several research studies that support the exogenous application of proline to improve stress tolerance. In water-stressed maize (*Zea mays* L.), a foliar spray with Pro improved plant growth and ameliorated the negative effects of water deficit (Ali et al., 2007, 2008). The exogenous application of proline was capable of mitigating the negative effects of drought in barley (*Hordeum vulgare*) in vegetative state (Abdelaal et al., 2020a), reaching higher levels of relative water content under stress conditions. In a previous work (Abdelaal et al., 2020b) studied salinity stress in sweet pepper (*Capsicum annuum*), showing that Pro was capable of doubling production in saline conditions. Furthermore, pre-sowing wheat seeds (*Triticum aestivum* L.) with 40 mM proline was the most effective treatment to enhance growth and yield under water stress (Kamran et al., 2009). Proline also improved salt tolerance with both foliar spray and root treatment in *Vigna radiata* (Hossain et al., 2011), rice (*O. sativa* L.) (Roy et al., 1993), *Vicia faba* L. (Gadallah, 1999) and tomato (*Lycopersicon esculentum* L.) (Heuer, 2003). It was also effective against freezing damage in spinach (*Spinacia oleraceae*) (Shin et al., 2018) and against oxidative stress in *Vitis vinifera* L. (Ozden et al., 2009). Several authors reported that the exogenous application of Pro improved growth and photosynthetic capacity, and Ali et al. (2008) observed that it promoted the uptake of essential nutrients. In a recent study (Hanif et al., 2020) showed that a foliar spray with Pro increased the activities of antioxidant enzymes, enhancing tolerance to drought and heat stress. Hoque et al. (2007) described a similar effect in tobacco cells. However, the protective action was observed only at low concentrations, since higher doses had toxic effects (Hayat et al., 2012).

Arginine (Arg) application has been shown to alleviate the harmful effects of salt stress in mung beans and canola plants (Qados, 2010; Nasibi et al., 2014), as well as oxidative stress caused by nickel accumulation in black henbane (*Hyoscyamus niger;* Nasibi et al., 2013) and by drought stress in tomato (Nasibi et al., 2011). Arg treatments are beneficial for the growth and development of maize plants, especially under cold stress conditions (Matysiak et al., 2020).

Cysteine (Cys) is capable of increasing soybean production after daily watering with sea water (Sadak et al., 2020b). Cys is a key precursor of the antioxidant tripeptide glutathione (GSH), which has an important role in protection against oxidative stress and heavy metal detoxification (Romero et al., 2014; Teixeira et al., 2017).

**FIGURE 4:**
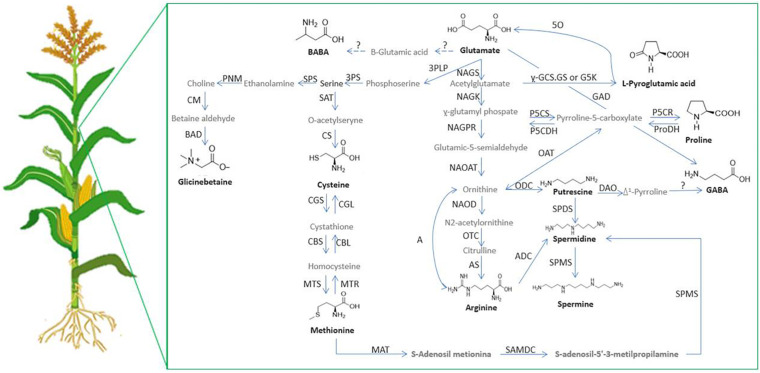
Relationships between proteinogenic/non-proteinogenic amino acids and polyamines in plants. 3PLP, 3-phosphoserine aminotranferase; 3PS, P-serine phospatase; 5O, oxoprolinase; A, arginase; ADC, arginine decarboxylase; AS, argininosuccinate synthase; BAD, betaine aldehide deshidrogenase; CBL, cystathionine β-lyase; CM, choline monooxigenase; CS, cysteine synthase; CGL, cysthathionine-y_._-lyase; CGS, cystathionine y_._-synthase;CBS, cystathionine β-synthase; DAO, diamine oxidase; y_._-GCS, y_._-glutamylcysteine synthetase; GAD, glutamate decarboxylase; GS, glutamine synthetase; G5K, glutamate 5-kinase; MAT, methionine adenosine transferase; MTS, methionine synthase; MTR, methyonine reductase; NAGK, N-acetylglutamate kinase; NAGPR, N-acetylglutamate-5-phosphate reductase; NAGS,N-acetylglutamate synthase; NAOAT, N-acetylornithine aminotransferase; NAOD, N-acetylornithine deacetylase; OAT, ornithine δ-aminotransferase; ODC, ornithine decarboxylase; OTC, ornithine transcarbamoylase; ProDH, proline dehydrogenase; P5CDH, Δ1-pyrroline-5-carboxylate dehydrogenase; P5CR, Δ1-pyrroline-5-carboxylate reductase; P5CS, Δ1-pyrroline-5-carboxylate synthetase; PNM, phosphoethanolamine N-methyltransferase; SAMDC, S-adenosylmethionine decarboxylase; SAT, Serine *O*-cetyltranserase; SPDS, spermidine synthase; SPS, serine-phosphoethanolamine synthase; PAO, polyamine oxidase; SPMS, spermine synthase.

In addition to the effects mentioned before, amino acids can act in plants as precursors of other amino acids or other defense compounds (Figure 4). Glu is a precursor of the antioxidant peptide glutathione, and also of other stress-related amino acids, such as proline. Proline biosynthesis from Glu involves a high consumption rate of NADH and NADPH. When energy is needed, proline oxidation would yield 30 ATP molecules. Therefore, proline reserves would be valuable either in acclimation to stress or for recovery after stress relief (Kaur and Asthir, 2015). In another example, Arg is an important amino acid for nitrogen storage in plants, and its catabolism mobilises stored nitrogen, which is involved in the production of NO, polyamines and potentially proline (Winter et al., 2015).

Amino acids seem to be effective in protecting plant growth under stress. The presented proteinogenic amino acids (Met, Glu, Pro, Arg, Cys) were the most referenced, since research on the other 15 was scarce or non-existent. Therefore, more studies are required on the others to verify their effectiveness. Furthermore, most of the studies reporting the effects of pure amino acids do not describe production results, with the exception of the studies with proline (Abdelaal et al., 2020b). Therefore, more production trials would be needed to fine-tune new commercial BS formulations.

### Non-protein Amino Acids

In addition to the amino acids found in proteins, plants contain other non-proteinogenic amino acids known as npaa, which are widely distributed in the Plant Kingdom. It has been reported that a large number of stresses can trigger the biosynthesis of npaa in Monocots and Eudicots. Among them, stresses induced by UV-radiation, salinity, hypoxia, heat, cold, drought, and heavy metals (Rodrigues-Corrêa and Fett-Neto, 2019). The npaa improve plant stress tolerance through cellular osmoregulation, enhancement of antioxidant defense systems, protection of membrane integrity, and enzyme/protein stabilisation (Yancey, 1994; Bohnert and Jensen, 1996; Ashraf and Foolad, 2007; Hayat et al., 2012).

Glycine-betaine (GB) is probably the main compatible osmolyte other than proline. Foliar and root treatment with this molecule has counteracted the effect of salt and drought stress in different species such as kidney bean (*Phaseolus vulgaris* L; Sofy et al., 2020), barley (*Hordeum vulgare* L.; Wang N. et al., 2019), broadbean (*V. faba* L.; Gadallah, 1999), corn (*Zea mays*, Ali and Ashraf, 2011), tomato (*S. lycopersicum*, Heuer, 2003) and lettuce (*Lactuca sativa*; Shams et al., 2016). Additionally, Ali and Ashraf (2011) obtained better quality and yield of maize seed and oil after applying GB. Furthermore, in cotton plants (*Gossypium* sp.) subjected to drought stress, GB application did not affect yield components, physiological processes, or endogenous GB levels (Meek et al., 2003). However, there are a few reports that describe counterproductive or no effects of GB on plant growth under stress conditions. This is the case of tomatoes, where 1 and 5 mM GB counteracted the effects of salt stress but inhibited plant growth (Heuer, 2003). This emphasises the need to understand GB mechanism of action, its effect on different plant species and phenological state, and dose-dependent effect.

γ-Aminobutiric acid (GABA) is ubiquitous in the plant kingdom and accumulates rapidly when plants are exposed to stress conditions (Bown and Shelp, 2016). Under drought stress, sprayed GABA increased drought tolerance in creeping bentgrass (*Agrostis stolonifera*) by enhancing the osmoregulatory metabolism, energy production and synthesis of secondary metabolites (Li et al., 2017a). It was also effective in maize subjected to salt stress, increasing photosynthesis, chlorophyll fluorescence, antioxidant activity and proline accumulation (Wang et al., 2017). Exogenous treatment with GABA increased muskmelon (*Cucumis melo*) tolerance to saline-alkaline stress (Jin et al., 2019), reducing the Na^+^/K^+^ ratio and increasing the concentration of free polyamines. Besides, GABA conferred tolerance to chromium stress on brown mustard (*Brassica juncea* L.), by enhancing its antioxidant defences (Mahmud et al., 2017).

β-aminobutyric acid (BABA) is a molecule related to GABA, which was initially considered as a synthetic priming agent. However, Thevenet et al. (2017) found that BABA is naturally generated in *Arabidopsis thaliana* under stress. BABA has been extensively studied, since it can induce resistance to several types of stress. Abid et al. (2020) demonstrated that BABA treatment is capable of inducing drought tolerance in *V. faba* L. by increasing the transcription of appropriate genes. In addition, seed priming of *V. radiata* enhanced its tolerance to salt and polyethylene glycol (PEG) stress, by increasing photosynthetic activities, antioxidant defences, and proline accumulation, and by reducing malondialdehyde content (Jisha and Puthur, 2016). BABA-enhanced tolerance to drought stress in maize is effected through the jasmonic acid (JA) pathway through the activation of antioxidant defences; abscisic acid (ABA) is also involved (Shaw et al., 2016). This is in agreement with Baccelli and Mauch-Mani (2016), who summarised the various defence signalling pathways potentiated by BABA, depending on the plant and stress applied. Interestingly, even though BABA increased wheat tolerance to soil drying, it did not affect grain yield (Du et al., 2012). This effect could be related to the microbial metabolism of BABA, and the resulting increase in abscisid acid.

Interestingly, although there are 10 times more npaa than proteinogenic amino acids, only a few npaa have been studied as BSs against abiotic stress (Rodrigues-Corrêa and Fett-Neto, 2019). This group of organic compounds could be a great source of new BSs. However, to assess their effectiveness, production measures should be carried out. As mentioned before for GB, Ali and Ashraf (2011) with maize and (Meek et al., 2003) with cotton, studied BS performance in crop production, reporting opposite results. On the other hand, Du et al. (2012) reported that BABA treatment increased tolerance to desiccation and decreased water use in spring wheat cultivars, but did not improve grain yield. However, other crops could provide different production results, and thus research on this topic should be promoted.

### Polyamines

Polyamines are biogenic amines involved in several functions in plants, such as growth, seed germination, flower and fruit development, cell division and elongation, membrane and cell wall stabilisation, and processes of replication, transcription and translation (Hussain et al., 2011). Putrescine (Put), spermidine (Spd), and spermine (Spm) are the main examples in plants (Berberich et al., 2015). These polyamines are osmoprotectors and potent BSs that activate the response to biotic and abiotic stress.

Foliar treatment with Put in common thyme plants (*Thymus vulgaris* L.) improved growth and oil yield under water stress (Abd Elbar et al., 2019). Furthermore, it showed protection against drought in wheat (Arslan et al., 2019) and lettuce (Zhu et al., 2019). In addition, foliar treatment of salt-tolerant rice under salt stress increased shoot growth, grain yield and proline content, inhibited Na^+^ and Cl^–^ uptake, and prevented chlorophyll degradation (Krishnamurthy, 1991). It also improved salt tolerance of yellow guava seedlings (*Psidium guajava* L.) (Esfandiari Ghalati et al., 2020). The exogenous application of Put also proved to be very effective in increasing the growth, photosynthetic pigments, yield and quality of onions (*Allium cepa*), offering better results than the application of Glu. However, it should be noted that the combination of Put and Glu gave the best results (Amin et al., 2011). Moreover, (Kim et al., 2002) reported the protective role of putrescine under cold stress. Thus, tomato plants treated with an inhibitor of putrescine synthesis increased their electrolyte leakage under cold stress, but exogenous application of putrescine reduced it.

Spermine (Spm) was used in foliar treatments to protect wheat from drought, providing slightly better results than Put (Hassan et al., 2020). However, the authors found that the combination of both polyamines was the most effective treatment, achieving protection through ROS removal, activation of the CAT enzyme, and improvement of the Na^+^/K^+^ ratio. Also, Spm improved the fresh weight and protein content of soybean pods and seeds under osmotic stress, as well as their antioxidant defences, and induced changes in ABA synthesis (Radhakrishnan and Lee, 2013). This polyamine also increased protection against salt stress in tomato seedlings by increasing the accumulation of osmolytes and secondary metabolites, as well as the activity of antioxidant system (Ahanger et al., 2019).

Exogenous Spd protected against drought and promoted grain filling in wheat, regulating its starch and antioxidant systems (Li et al., 2020). Furthermore, foliar treatment with Spd improved tolerance to moderate salt stress in pecan-grafted seedlings by increasing the activity of antioxidant enzymes, reducing the Na^+^/K^+^ ratio, and suppressing the induction of ABA and ethylene (Wu Z. et al., 2020). In a previous work, Roy et al. (2005) found that it protected against salt stress by impeding the inhibition of plasma membrane-bound H^+^-ATPase, which acts as a pump involved in K^+^/H^+^ exchange. In addition, the authors found higher levels of plasma membrane-bound Spd and Spm, as well as H^+^-ATPase pumps in salt-tolerant rice, which appear to stabilise the plasma membrane by keeping endogenous Na^+^ levels low and K^+^ levels high.

In the polyamine biosynthesis pathway, there are two amino acids involved. Arg is the precursor to Put in three biogenic routes, and then Met provides aminopropyl residues to produce Spd and Spm from Put (Chen et al., 2019). The catabolism of polyamines is also interesting, since it produces H_2_O_2_ which at low concentrations can act as a stress signalling molecule that induces a ROS-dependent protective pathway (Wang W. et al., 2019).

Therefore, the protective roles of polyamines have been studied extensively, including: (i) as osmoprotectans; (ii) acting as ROS scavengers and increasing the production of antioxidant enzymes; (iii) Interact with DNA, RNA and the transcriptional complex, as well as with cell and organellar membranes; (iv) as signal molecules by themselves or through the production of H_2_O_2_ in ABA-regulated stress response pathway; (v) regulation of ion channels and (vi) role in programmed cell death (Minocha et al., 2014). This knowledge facilitates progress toward field trials to discover how Pas can increase production.

### Biopolymers

Biopolymers are polymers synthesised by living organisms. There are some interesting groups, such as polypeptides or polysaccharides (polymeric carbohydrates). Some of these polymers can be used as pure organic active compounds against abiotic stress, and are commented below.

Chitosan, the most abundant polymer after cellulose, and its oligomers (oligochitosan), are linear polysaccharides formed by β-(1-4)-linked D-glucosamine and N-acetyl-D-glucosamine. Chitosan elicited much interest due to its effect against biotic and abiotic stress, besides being environmentally friendly and inexpensive (Katiyar et al., 2015). Regarding stress tolerance, (Rabêlo et al., 2019) reported that chitosan is capable of increasing tolerance to stress due to water deficit in maize. Interestingly, this treatment improved antioxidant systems, photosynthesis and grain yield. In barley, chitosan also improved the response to drought stress (Hafez et al., 2020).

Zeng and Luo (2012) found that coating wheat seedlings with chitosan improved drought tolerance by influencing physiological mechanisms, such as increasing antioxidant defences and improving chlorophyll content. This allowed for better plant growth and root development. The authors also found that the treatment enhanced seed germination and yield. The study by Li et al. (2017b) should be highlighted, since a metabolomic analysis of white clover treated with chitosan under drought stress revealed that this biopolymer increased the accumulation of several osmoprotectants related to antioxidant defence and stress signalling. Chitosan was also effective against water stress in *Thymus daenensis* Celak (Bistgani et al., 2017) and two species of basil (*Ocimum ciliatum* and *Ocimum basilicum*) (Pirbalouti et al., 2017). In the same way, it protected against cadmium (Cd) toxicity in *Brassica rapa* L. plants (Zong et al., 2017a, b) and against the effects of ozone in rice (Phothi and Theerakarunwong, 2017).

Alginate oligosaccharides (AOS), polymers that are obtained from marine brown algae, present advantages such as relative low cost, low toxicity, mild gelation, and biocompatibility (Lee and Mooney, 2012). They have proved to be promising BSs to increase plant stress tolerance. Indeed, AOS enhanced tolerance to PEG-induced drought stress in wheat (Liu et al., 2013), tomato seedlings (Liu et al., 2009), and to drought stress in potted cucumber (Li et al., 2018) and also Cd tolerance in wheat (Ma et al., 2010). In the PEG-induced stresses, AOS increased biomass and antioxidant enzymes. Liu et al. (2013) reported an increase in proline and chlorophyll content, and recently, Li et al. (2018) commented that alginates promoted expression of drought resistance genes and regulated ABA-dependent signal transduction.

Poly (γ-glutamic acid) (γ-PGA) is a polypeptide composed of D- and L-glutamic acid monomers which is generated by microbial fermentation (Shih and Van, 2001). The polypeptide has promising properties, such as its biodegradability, non-toxicity, water solubility and low production cost (Chen et al., 2005). Xu et al. (2020) define γ-PGA as an anti-drought agent that can efficiently alleviate damage to plants under drought stress by promoting the accumulation of abscisic acid in rape (*Brassica napus* L.) as well as increasing enzymatic antioxidant activity and accumulation of proline. In addition, the polymer is also effective against salt and cold stress in rape (Lei et al., 2016; Xu et al., 2017), by enhancing proline accumulation. Besides, (Xu et al., 2017) reported that pretreatment with γ-PGA induced cross-talk between Ca^2+^, H_2_O_2_, brassinolide and jasmonic acid, resulting in the accumulation of proline and the improvement of the antioxidant machinery. On the other hand, in wheat seedlings, γ-PGA increased antioxidant defences and modulated ionic balance (Guo et al., 2017). It also protected garden cucumber (*Cucumis sativus* L.) seedlings against Cd and Pb toxicity (Pang et al., 2018). The effect of the polypeptide γ-PGA on soil moisture and microbial communities has recently been reported. It improved drought resistance of maize seedlings by improving soil moisture and nutrient levels, stimulating plant growth-promoting bacteria, and reducing pathogenic fungi (Yin et al., 2018).

Some microbial biopolymers also deserve attention (for uses other than abiotic stress, see the review by Naamala and Smith, 2020). Thus, direct inoculation of lipochitooligosaccharides (LCOs) and/or thuricin-17 peptide, compounds described as bacterial signals, can protect plants against different abiotic stresses (Nazari and Smith, 2020). For instance, when soybean seeds were treated with both compounds, they were more resistant to high salt stress (Subramanian et al., 2016). Moreover, in experiments in growth chambers (Prudent et al., 2015) showed that when soybean plants associated with N2-fixing B. japonicum received a root application of thuricin17, their resistance to drought increased. However, more studies and specific field trials are needed to assess their effectiveness for crop production.

**FIGURE 5:**
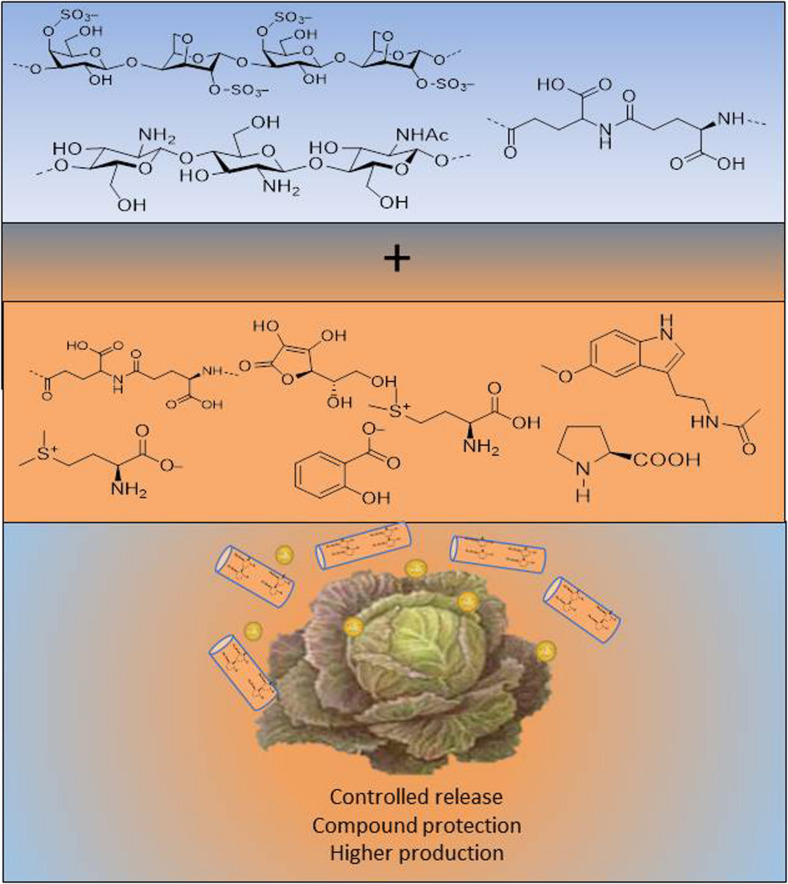
Benefits of nanoencapsulation of biopolymers.

Finally, it should be pointed out that alginates and also chitosan, are used to encapsulate pesticides and fertilisers, but this issue has not been well studied with BSs (Jiménez-Arias et al., 2020). However, Juárez-Maldonado et al. (2019) proposed nanoparticles and nanomaterials as a new category of biostimulant. Both individual polymers and nanoparticle derivatives with encapsulated products would open up promising strategies to improve field production (Figure 5).

### Vitamins

Vitamins are classified as fat-soluble or water-soluble. While the former display important antioxidant properties, the latter normally serve as enzyme cofactors (Hanson et al., 2016). Both types have important functions in plants and their exogenous application can enable a greater tolerance to several stresses.

A notable case is ascorbic acid (vitamin C), which is an important metabolite in plants with several functions, such as co-factor of enzymes or modulator of plant defences by detoxification of hydrogen peroxide (Hemavathi et al., 2011; Zhang et al., 2012; Mostofa et al., 2015; Akram et al., 2017). For this reason, exogenous application of vitamin C proved to be effective in protecting several plant species against drought stress. The assays were carried out in the grass “tall fescue” (*Festuca arundinacea* Schreb, Xu et al., 2015), in wheat (*T. aestivum* L., Hafez and Gharib, 2016) and safflower (*Carthamus tinctorius* L., Farooq et al., 2020). The findings of Aziz et al. (2018) are also interesting, since they tested the effects of pure synthetic ascorbic acid and natural sweet orange juice extract as a natural source of vitamin C, in quinoa (*Chenopodium quinoa*) under drought stress. They found that both treatments were effective in improving tolerance to this stress, although in some cases orange juice was more effective. The authors supposed that this result was due to the variety of molecules and nutrients present in orange juice that may enhance the stress response more than ascorbic acid alone. Treatment with ascorbic acid was also found to increase the growth and seed yield of common bean (*Phaseoulus vulgaris*, Gaafar et al., 2020) and broadbean (*V. faba* L., Desoky et al., 2020) under water stress. In another study, the tolerance to drought stress of pepper plants (*C. annuum* L) increased with ascorbic acid treatment, by increasing the activity of antioxidant enzymes, but growth and yield were affected compared to those achieved with full irrigation (Khazaei et al., 2020).

Vitamin C was also reported to be effective against salt stress in tomato, and its protective role is probably related to its antioxidant properties, since it reduced lipid peroxidation but not sodium uptake and plasma membrane leakiness (Shalata and Neumann, 2001). A field experiment with sugar beet (*Beta vulgaris* var. saccharifera, L.) under salt stress showed that soaking the seeds plus foliar spraying with ascorbic acid increased the enzymatic activity of CAT and SOD, as well as root yield and sugar content (Abdel Fatah and Sadek, 2020). In another work, both vitamin C and B_3_ alleviated the effects of salt stress, but vitamin B_3_ was more effective, and in combination they were synergistic (Azooz et al., 2013).

α-Tocopherol is the most abundant vitamin E compound in photosynthetic tissues. It protects the lipid membranes by preventing the propagation of lipid peroxidation by quenching/scavenging of reactive oxygen species (ROS; Miret and Munné-Bosch, 2015). Treatment with vitamin E of chinese rye grass (*Leymus chinensi*) seedlings subjected to PEG stress enhanced the activity of the peroxidases SOD and POD. It also increased proline content, and reduced lipid peroxidation (Gu et al., 2008). In another study, the foliar application of α-tocopherol was effective in increasing wheat tolerance to drought, by improving antioxidant defence mechanisms, water use, photosynthetic efficiency and the content of photosynthetic pigments (Ali et al., 2019). The authors also found that the treatment increased wheat biomass, as well as the yield and nutritional quality of the seeds.

A scarcely studied vitamin is S-methylmethionine (vitamin U). It is produced by all angiosperms, since it is involved in their sulphur metabolism (Ludmerszki et al., 2014; Fodorpataki et al., 2019). In fact, its role in the biosynthesis of sulfopropionates (osmoprotectants) and polyamines is valuable for plant resistance (Ludmerszki et al., 2014). It was highly effective in protecting maize against cold stress, by stimulating the phenylpropanoid pathway, increasing the content of phenol derivatives and anthocyanins, and protecting the photosynthetic apparatus (Páldi et al., 2014). Priming lettuce with this vitamin resulted in a greater tolerance to cold, improving its germination, photosynthetic efficiency, and content of carotenoids and vitamin C (Fodorpataki et al., 2019).

S-methylmethionine-salicylate (MMS) has been synthesised from vitamin U and salicylic acid, and benefits from both protective roles. It was tested in wheat plants under salt stress, and compared with vitamin U and salicylic acid (Janda et al., 2018). All three compounds were harmful at 0.5 mM but at a lower concentration (0.1 mM) they were innocuous, while protecting against stress. In this study, the protective action of MMS did not correspond to a synergistic effect of vitamin U and salicylic acid, since they presented similar modes of action. Other results that support this hypothesis were obtained with maize under cold stress (Páldi et al., 2014; Oláh et al., 2018).

Menadione sodium bisulphite is a chemical modification of vitamin K_3_ which increases tolerance to salt stress in *Arabidopsis* after seed treatment (Jiménez-Arias et al., 2015b) and in tomato plants by root treatment (Jiménez-Arias et al., 2019b). Thus, the authors found that MSB produces a slight oxidative burst that triggers plant defences, facilitating a higher relative growth rate, photosynthesis and other gas-exchange parameters. It also produced epigenetic changes in the promoter region involved in proline metabolism, increasing the proline content (Jiménez-Arias et al., 2015a). Menadione sodium bisulphite was also able to enhance antioxidant defences and the expression of proteins regulating Na^+^ and K^+^ levels, which improved the ionic homeostasis under salt stress (Jiménez-Arias et al., 2019b).

Most of the work on the exogenous application of vitamins focuses on ascorbic acid, and studies on its performance are increasing. On the other hand, the protective role of α-tocopherol in plants is well known, but there is little work on its exogenous application. Due to their properties, vitamins are excellent options for research on crop protection and productivity. In addition, new studies are being carried out on vitamin derivatives.

### Melatonin

This compound is a multifunctional molecule distributed in different parts of plants and involved in several physiological processes: the circadian rhythm, photosynthesis, biomass production, root development, seed germination, fruit ripening, foliar senescence, membrane integrity, redox network, osmoregulation and response to abiotic stress (Khan et al., 2020). Several works have shown that exogenous application of melatonin can increase plant growth and resistance to stress. Therefore, melatonin has been proposed as a natural biostimulant for a sustainable and eco-friendly agriculture.

The exogenous application of melatonin to maize seedlings under drought stress reduced the accumulation of hydrogen peroxide and malondialdehyde, and increased photosynthesis, transpiration and stomatal conductance (Ye et al., 2016). Xia et al. (2020) found that treatment with melatonin alleviated drought stress in kiwi seedlings, by increasing the level of mRNA expression of the enzymes SOD, CAT, and peroxidase, and enhancing their activity. Moreover, three pathways implicated in melatonin protection were reported: the ascorbate and aldarate metabolism, glutathione metabolism and carotenoid metabolism. These pathways were involved in higher levels of ascorbic acid, glutathione and carotenoids. Melatonin also alleviated oxidative stress caused by water stress in two species of *Salvia* and increased the essential oil production (Bidabadi et al., 2020). In addition, the foliar application of melatonin in moringa trees (*Moringa oleifera* L.) under both normal irrigation and drought in a field trial, increased tree growth and its oil yield and quality (Sadak et al., 2020a).

Melatonin was also effective in protecting cucumber seedlings against salt stress, by enhancing the activity of antioxidant enzymes, inducing gene expression related with salt stress, and protecting the photosynthesis (Zhang et al., 2020). Treatment with melatonin also increased the fruit yield and quality in strawberry (*Fragaria* x *ananassa* Duch.), especially in plants under salt stress (Zahedi et al., 2020). The study found that melatonin boosted the activity of leaf antioxidant enzymes, as well as ABA and melatonin contents in leaf and fruits. Therefore, it was proposed that melatonin induced antioxidant defences by ABA-dependent and independent signalling pathways.

Melatonin also effectively protected melon roots against cooper stress (Hu et al., 2020), strawberry seedlings against Cd stress (Wu S. et al., 2020), and tomato seedlings against nickel stress (Jahan et al., 2020). In addition, it protected cherry radish (*Raphanus sativus* L.) against high temperature (Jia et al., 2020), and tomato plants (*S. lycopersicum*) against chilling (Wang M. et al., 2020). It was suggested that the protective role of melatonin is due to the regulation of the antioxidant defence system, from direct ROS scavenging by reaction with H_2_O_2_, O_2_^–^, and OH^⋅^, to an increase in the activity of antioxidant enzymes (CAT, SOD, and GPX), as well as enzymes of the ascorbate-glutathione cycle (ascorbate peroxidase, monodehydroascorbate reductase, dehydroascorbate reductase, and glutathione reductase). It is also involved in the effects of non-enzymatic antioxidants (carotenoids, tocopherols, ascorbate, reduced glutathione, and phenolic compounds) (Khan et al., 2020).

Although the positive effects of melatonin application are well established, most studies have been conducted under controlled conditions. Therefore, Khan et al. (2020) highlighted the need for more field trials and transcriptomics analysis. On the other hand, Wang S.-Y. et al. (2020) reported an increasing trend in studies to improve fruit yield and quality, and to enhance resistance.

## Conclusion and Future Perspectives

Pure organic active compounds offer several benefits, such as better standardisation and quality control of formulations, and a better understanding of their protective action, including not only the mode but also the mechanism of action. This will allow a better design of new formulations, either with a pure active principle or with a precisely dosed mixture of synergistic compounds. Additionally, pure compounds or carefully selected combinations can shed light on many effects of BSs, aided by molecular biology techniques (including transcriptomics, metabolomics, proteomics, and genomics) and ever-growing bioinformatic analyses (Bulgari et al., 2019).

**FIGURE 6:**
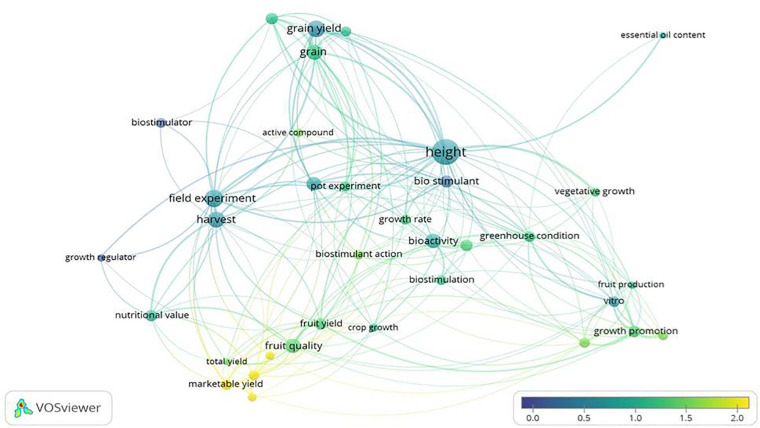
Map of terms of the publications on biostimulants. The words “biostimulant” and “plant” were searched in the titles, abstracts and keywords, during the period from 2010 to 2020. The analysis is based on all the publications that are classified as Articles. The distance between terms indicates how strong their relationship is. The colour represents the normalised citation average, where blue is a low citation average and yellow is a high citation average. The size indicates the number of publications in which the term appears. References taken from WoS and Scopus.

Some interesting data on the impact of biostimulant research can be drawn from the analysis of titles and abstracts of publications, using the VOSviewer software (Figure 6). The terms that appeared most frequently were: “height,” “grain yield,” “field experiment,” and “harvest,” of which the latter two were closely related. However, these terms had low-medium average citations. The high incidence of the term “height” highlights that it is a fairly common measure in BSs studies. On the other hand, “marketable yield” and “fresh yield” were also highly cited, while “fruit quality” and “fruit yield” presented medium averages of citations, but low occurrence in the studies. In contrast, studies on cereal/grain yield are common but had less average citations.

Thus, it seems that there is a need for more field trials as well as studies on the yield and quality of crops, particularly fruits. As can be seen from the Table in the Supporting Information, most studies have been carried out in culture chambers or greenhouses. The reason is that field experiments present challenges because they require more space, time, and resources. In addition, variable weather conditions make it compulsory to repeat farming cycles to guarantee reproducible results. Nevertheless, it is necessary to confirm in field trials that the BS (as a pure organic active compound or as a product mixture) increases crop yield without loss of nutritional value, since the laboratory studies carried out in vegetative growth stage do not provide information on these key issues. Therefore, field results and impact on production could be a wellcome addition to the preliminary (and often promising) results.

Furthermore, several studies have applied severe stress in both laboratory and field conditions. Although these studies are useful for identifying individual compounds with a significant protective effect, as mentioned before, Serraj and Sinclair (2002) emphasise that many of these results have little practical value for farmers. This is because studies carried out under severe stress focus on plant survival, even though the yields thus obtained are too low to be viable for the industry. To address this problem, some authors use more moderate conditions, closer to real scenarios. Thus, a recent study on drought-stressed lettuce only reduced normal irrigation by 30%, with promising results (Jiménez-Arias et al., 2019a). Lettuce is an excellent model for this stress since it is sensitive to drought and the yield is based on vegetative growth. Similar studies with fruit crops would be desirable.

It is interesting to compare the costs and benefits of using pure organic active compounds to increase yield under stress conditions. Thus, from 1983 to 2009, three-quarters of harvested areas globally suffered yield losses caused by drought, entailing 166 billion US dollars of cumulative production losses (Kim et al., 2019). However, the application of α-tocopherol increased 556.5 kg/ha of wheat yield under water deficit, which means a benefit of US $ 231.80 (Ali et al., 2019). This highlights that a pure organic active compound could be a sustainable solution to face losses caused by abiotic stress and increase economic benefits in agriculture. In addition, BSs could be valuable to achieve better nutritional and organoleptic qualities of crop products, that increase their value. The potential in this issue of pure compounds, alone or in carefully designed combinations, should receive more attention in future.

## Author Contributions

AG-G elaborated the information, and prepared the Figures and Schemes. The review was completed by the other coauthors in their research areas, and corrected by the corresponding authors, who also polished text and figures and added information to the initial versions. All the authors contributed in browsing recent literature on the subject.

## Conflict of Interest

The authors declare that the research was conducted in the absence of any commercial or financial relationships that could be construed as a potential conflict of interest.
