# Supplementary material for: Pure Organic Active Compounds Against Abiotic Stress: A Biostimulant Overview
Source: Front Plant Sci. 2020 Dec 23;11:575829. doi: 10.3389/fpls.2020.575829 (PMC7785943; doi:10.3389/fpls.2020.575829)
Supplement: Supplementary file 1 [file Table_1.DOCX]

Table 1. Experimental conditions under the pure organic active compounds where tested and the species studied. Uncontrolled conditions refer to different places than field, greenhouse or growth chamber that could be opened or closed, or where the conditions are not clear in the reference.

| Compounds | Specie | Conditions | Stress | Production | Reference |
| --- | --- | --- | --- | --- | --- |
| Amino acids and other *N*-containing compounds |  |  |  |  |  |
| - Proteinogenic amino acids |  |  |  |  |  |
| Methionine | *Vigna unguiculata* L. | Greenhouse | Drought | Not evaluated | Merwad et al., (2018) |
|  | *Solanum lycopersicum* | Growth chamber | Salt | Not evaluated | Alfosea-Simón et al., (2020) |
|  | *Momordica charantia* L. | Uncontrolled conditions | Drought | Not evaluated | Akram et al. (2020) |
| Glutamate | *Oryza sativa* L. | Growth chamber | Cold | Not evaluated | Jia et al. (2017) |
|  | *Allium cepa* L. | Field | Salt | 200 ppm increase yield | Amin et al. (2011) |
|  | *Glycine max* L. | Greenhouse and field | Drought | Not evaluated | Teixeira et al. (2017) |
|  | *Brassica napus* | Greenhouse | Drought | Not evaluated | La et al. (2020) |
| Proline | *Zea mays* L*.* | Greenhouse | Drought | Not evaluated | Ali et al. (2007, 2008) |
|  | *Hordeum vulgare* | Field | Drought | 10 mM proline increase yield | Abdelaal et al. (2020a) |
|  | *Capsicum annuum* | Uncontrolled conditions | Salt | Not evaluated | Abdelaal et al. (2020b) |
|  | *Triticum aestivum* L. | Uncontrolled conditions | Drought | Not evaluated | Kamran et al. (2009) |
|  | *Vigna radiata* | Growth chamber | Salt | Not evaluated | Hossain et al. (2011) |
|  | *Oryza sativa* L. | Growth chamber | Salt | Not evaluated | Roy et al. (1993) |
|  |  | Greenhouse | Drought | 30 mM proline increase yield | Hanif et al. (2020) |
|  | *Vicia faba* L. | Uncontrolled conditions | Salt | Not evaluated | Gadallah, (1999) |
|  | *Lycopersicon esculentum* L. | Growth chamber | Salt | Not evaluated | Heuer (2003) |
|  | *Spinacia oleraceae* | Greenhouse | Cold | Not evaluated | Shin et al. (2018) |
|  | *Vitis vinifera* L. | Growth chamber | Oxidative | Not evaluated | Ozden et al. (2009) |
| Arginine | *Vigna radiata* | Greenhouse | Salt | Not increase yield | Qados (2010) |
|  | *Brassica napus* | Uncontrolled conditions | Salt | Not evaluated | Nasibi et al. (2014) |
|  | *Hyoscyamus niger* | Uncontrolled conditions | Nickel | Not evaluated | Nasibi et al. (2013) |
|  | *Zea mays* L. | Growth chamber, greenhouse | Heat | Not evaluated | Matysiak et al. (2020) |
| Cysteine | *Glycine max* L. | Greenhouse | Salt | 20 ppm increase yield | Sadak et al. (2020) |
| - Non-protein amino acids (Npaa) |  |  |  |  |  |
| Glycine-betaine | *Phaseolus vulgaris* L. | Field | Salt | Not evaluated | Sofy et al., 2020 |
|  | *Hordeum vulgare* L. | Greenhouse | Drought | Not evaluated | Wang et al. (2019a) |
|  | *Vicia faba* L. | Uncontrolled conditions | Salt | Not evaluated | Gadallah (1999) |
|  | *Zea mays* L. | Field | Drought | Not evaluated | Ali and Ashraf (2011) |
|  | *Solanum lycopersicum* | Growth chamber | Salt | Not evaluated | Heuer (2003) |
|  | *Lactuca sativa* | Greenhouse | Salt | Not evaluated | Shams et al. (2016) |
|  | *Gossypium* sp | Field | Drought | Not increase yield | Meek et al., 2003 |
| γ-Aminobutiric acid | *Agrostis stolonifera* | Greenhouse | Drought | Not evaluated | Li et al. (2017a) |
|  | *Zea mays* L. | Greenhouse | Salt | Not evaluated | Wang et al. (2017) |
|  | *Cucumis melo* | Uncontrolled conditions | Salt | Not evaluated | Jin et al. (2019) |
|  | *Brassica juncea L.* | Growth chamber | Chromium | Not evaluated | Mahmud et al. (2017) |
| β-aminobutyric acid | *Vicia faba* L. | Growth chamber | Drought | Not evaluated | Abid et al. (2020) |
|  | *Zea mays* L. | Growth chamber | Drought | Not evaluated | Shaw et al. (2016) |
|  | *Triticum aestivum* L. | Greenhouse | Drought | Not increase yield | Du et al. (2012) |
| - Polyamines |  |  |  |  |  |
| Putrescine | *Thymus vulgaris* L. | Greenhouse | Drought | Not evaluated | Abd Elbar et al. (2019) |
|  | *Lactuca sativa* L. | Growth chamber | Drought | Not evaluated | Zhu et al. (2019) |
|  | *Oryza sativa* L. | Uncontrolled conditions | Salt | Not evaluated | Krishnamurthy (1991) |
|  | *Psidium guajava* L. | Nursery | Salt | Not evaluated | Esfandiari Ghalati et al. (2020) |
|  | *Allium cepa* | Field | Salt | 100 ppm increase yield | Amin et al. (2011) |
|  | *Solanum lycopersicum* | Growth chamber | Cold | Not evaluated | Kim et al. (2002) |
| Spermine | *Triticum aestivum* L. | Growth chamber | Drought | Not evaluated | Hassan et al. (2020) |
|  | *Glycine max* L. | Growth chamber | Drought | Not evaluated | Radhakrishnan and Lee (2013) |
|  | *Solanum lycopersicum* | Greenhouse | Salt | Not evaluated | Ahanger et al. (2019) |
| Spermidine | *Triticum aestivum* L. | Uncontrolled conditions | Drought | Not evaluated | Li et al. (2020) |
|  | *Carya* sp. | Greenhouse | Cadmium | Not evaluated | Wu et al. (2020b) |
|  | *Oryza sativa* L. | Growth chamber | Salt | Not evaluated | Roy et al. (2005) |
| Biopolymers |  |  |  |  |  |
| Chitosan | *Zea mays* L. | Greenhouse | Drought | 0.5 ppm increase yield | Rabêlo et al. (2019) |
|  | *Hordeum vulgare* L. | Field | Drought | 400 μL L^−1^ incresae yield | Hafez et al. (2020) |
|  | *Triticum aestivum* L. | Uncontrolled conditions | Drought | Not evaluated | Zeng and Luo (2012) |
|  | *Trifolium repens* | Growth chamber | Drought | Not evaluated | Li et al. (2017b) |
|  | *Thymus daenensis* Celak | Greenhouse | Drought | 400 μL L^−1^ incresae yield | Bistgani et al. (2017) |
|  | *Ocimum* sp. | Field | Drought | Not evaluated | Pirbalouti et al. (2017) |
|  | *Brassica rapa* L. | Growth chamber | Cadmium | Not evaluated | Zong et al. (2017a, 2017b) |
|  | *Oryza sativa* L. | Growth chamber | Oxidative | Not evaluated | Phothi and Theerakarunwong, (2017) |
| Alginate oligosaccharides | *Triticum aestivum* L. | Uncontrolled conditions | Drought | Not evaluated | Liu et al. (2013) |
|  |  | Growth chamber | Cadmium | Not evaluated | Ma et al. (2010) |
|  | *Solanum lycopersicum* | Growth chamber | Drought | Not evaluated | Liu et al. (2009) |
|  | *Cucumis sativus* L. | Uncontrolled conditions | Drought | Not evaluated | Li et al. (2018) |
| Poly (γ-glutamic acid) | *Brassica napus L.* | Growth chamber | Drought | Not evaluated | Xu et al. (2020) |
|  |  | Growth chamber | Salt | Not evaluated | Lei et al (2016) |
|  |  | Growth chamber | Salt/Cold | Not evaluated | Xu et al (2017) |
|  | *Triticum aestivum* | Growth chamber | Salt | Not evaluated | Guo et al (2017) |
|  | *Cucumis sativus L* | Growth chamber | Cadmium | Not evaluated | Pang et al (2018) |
|  | *Zea mays* L. | Greenhouse | Drought | Not evaluated | Yin et al (2018) |
| Lipochitooligosaccharides (LCOs) | *Glycine max* (L.) | Germination chamber | Salt | Not evaluated | Subramanian et al, 2016 |
| Thuricin-17 | *Glycine max* (L.) | Germination chamber | Salt | Not evaluated | Subramanian et al, 2016 |
|  |  | Growth chamber | Drought | Not evaluated | Prudent, Smith et al, 2015 |
| Vitamins |  |  |  |  |  |
| Ascorbic acid | *Festuca arundinacea* Schreb. | Growth chamber | Drought | Not evaluated | Xu et al, 2015 |
|  | *Triticum aestivum* L. | Field | Drought | 200 ppm increase yield | Hafez and Gharib, 2016 |
|  | *Carthamus tinctorius* L. | Uncontrolled conditions | Drought | Not evaluated | Farooq et al, 2020 |
|  | *Chenopodium quinoa* | Uncontrolled conditions | Drought | Not evaluated | Aziz et al, 2018 |
|  | *Phaseoulus vulgaris* | Field | Drought | Not evaluated | Gaafar et al., 2020 |
|  | *Vicia faba* L. | Field | Drought | 1.5 mM increase yield | Desoky et al. (2020) |
|  | *Capsicum annuum* L. | Greenhouse | Drought | Not evaluated | Khazaei et al. (2020) |
|  | *Solanum lycopersicum* | Growth chamber | Salt | Not evaluated | Shalata and Neumann (2001) |
|  | *Beta vulgaris* | Field | Salt | 200 ppm increase yield | Abdel Fatah and Sadek (2020) |
| α-Tocopherol | *Leymus chinensi* | Greenhouse | Drought | Not evaluated | Gu et al. (2008) |
|  | *Triticum aestivum* L. | Field | Drought | Not evaluated | Ali et al. (2019) |
| S-methylmethionine (vitamin U) | *Zea mays* L. | Growth chamber | Cold | Not evaluated | Páldi et al. (2014) |
|  | *Lactuca sativa* L. | Growth chamber | Cold | Not evaluated | Fodorpataki et al., (2019) |
| S-methylmethionine-salicylate (MMS) | *Triticum aestivum* L. | Growth chamber | Salinity | Not evaluated | Janda et al. (2018) |
|  | *Zea mays* L. | Growth chamber | Cold | Not evaluated | Oláh et al. (2018) |
| Menadione sodium bisulphite (MSB) | *Arabidopsis thaliana* | Growth chamber | Salt | Not evaluated | Jiménez-Arias et al. (2015a) |
|  | *Solanum lycopersicum* | Growth chamber | Salt | Not evaluated | Jiménez-Arias et al. (2019b) |
|  | *Arabidopsis thaliana* | Growth chamber | Salt | Not evaluated | Jiménez-Arias et al. (2015b) |
| Melatonin | *Zea mays* L. | Uncontrolled conditions | Drought | Not evaluated | Ye et al. (2016) |
|  | *Actinidia deliciosa* | Greenhouse | Drought | Not evaluated | Xia et al. (2020) |
|  | *Salvia* sp*.* | Greenhouse | Drought | 200 µM increase yield | Bidabadi et al. (2020) |
|  | *Moringa oleifera L.* | Field | Drought | 100 mM increase yield | Sadak et al. (2020) |
|  | *Cucumis sativus* L. | Growth chamber | Salt | Not evaluated | Zhang et al. (2020) |
|  | *Fragaria x ananassa* Duch. | Greenhouse | Salt | 100 µM increase yield | Zahedi et al. (2020) |
|  |  | Greenhouse | Cadmium | Not evaluated | Wu et al. (2020a) |
|  | *Cucumis melo* L. | Uncontrolled conditions | Copper | Not evaluated | Hu et al. (2020) |
|  | *Solanum lycopersicum* | Growth chamber | Nickel | Not evaluated | Jahan et al. (2020) |
|  |  |  |  |  |  |
|  | *Raphanus sativus* L. | Growth chamber | Heat | Not evaluated | Jia et al. (2020) |
